# Prenatal diagnosis and mRNA profiles of fetal tetralogy of Fallot

**DOI:** 10.1186/s12884-022-05190-0

**Published:** 2022-11-19

**Authors:** Zhongshan Gou, Yan Zhou, Hongjing Jia, Zhong Yang, Qian Zhang, Xinxin Yan

**Affiliations:** 1 Cardiovascular Disease Center, The Affiliated Suzhou Hospital of Nanjing Medical University, Jiangsu 215008 Suzhou, P.R. China; 2 Department of Ultrasonography, The Fourth Affiliated Hospital of Anhui Medical University, 23000 Hefei, Anhui P.R. China; 3 Department of Ultrasonography, The Affiliated Suzhou Hospital of Nanjing Medical University, 215008 Suzhou, Jiangsu P.R. China; 4 Department of Pharmacology, The Affiliated Suzhou Hospital of Nanjing Medical University, Jiangsu 215008 Suzhou, P.R. China

**Keywords:** Fetal tetralogy of fallot, Fetal echocardiogram, Autopsy, lncRNA, mRNA, Interactive effect

## Abstract

Tetralogy of fallot (TOF) in the fetus is a typical congential heart disease that occurs during the early embryonic period, being characterized by the abnormal development of conus arteriosus. The early diagnosis and prevention of fetal TOF is very important and there is a great need for exploring the pathogenesis of it in clinic. In this study, there were three cases being detected with TOF by fetal echocardiogram and confirmed by autopsy. We characterize the difference of expression of lncRNAs and mRNAs through sequencing analysis of 3 pairs of myocardial tissues of fetal TOF and those of age-matched controls. Compared with normal group, there were 94 differentially expressed lncRNAs and 83 mRNA transcripts in TOF (P < 0.05). Correlation analysis between lncRNA and mRNA further showed that differentially expressed lncRNA can be linked to mRNAs, suggesting the potential regulator role of lncRNA in mRNA expression. Our data serve as a fundamental resource for understanding the disease etiology of TOF.

**Supplementary Information:**

The online version contains supplementary material available at 10.1186/s12884-022-05190-0.

## Introduction

Tetralogy of fallot (TOF) in the fetus is a typical congential heart disease that occurs during the early embryonic period [1]. Of infants born with congenital heart disease, approximately 3.5% will have TOF, which is 0.28 per 1000, or 1 in 3600, live births [2]. The natural mortality of infant TOF even reaches up to 90% if the compromised cases has not been surgically corrected in time [3]. Males and females are equally affected. TOF occurs when the early embryo fails to follow the normal biology procedures (usually happens in the first eight weeks). Thus, the early diagnosis and prevention of fetal TOF is very important and enthusiasm has been raised to explore the etiology of TOF [4–6].

Previous studies reported that the occurrence of fetal TOF may be caused by genetic and/or environmental factors [7]. And there are no genetic anomalies identified in most cases with TOF [8]. However, in some cases, multiple genetic factors are considered to be relevant to the ocurrance of fetal TOF, or TOF appears as a manifestation of a genetic syndrome. In these cases, a few causative genes have been identified [9, 10]. Recent studies have found that abnormal expression of long noncoding RNAs (lncRNA) is closely associated with pathological physiology progression and prognosis of TOF [11, 12]. As a consequence, the role of lncRNA in investigating the molecule pathology mechanism of fetal TOF has been increasingly attentioned. lncRNAs are a class of RNA transcripts with a length of over 200 nucleotides which can regulate gene expression [13]. More and more evidences now suggest that lncRNAs are important for the development of heart. Compared with protein-coding mRNAs and other non-coding RNAs, lncRNAs have higher tissue specificity, which makes them more likely to be used as new diagnostic and prognostic biomarkers [14]. However, investigation of lncRNA in fetal TOF remains lacking.

In this study, we detailedly characterized the expression differences of lncRNAs and mRNAs in fetal myocardial tissues between TOF and normal fetuses, and further checked whether lncRNAs potentially regulate mRNA expression in a TOF depengdent manner, in order to provide fundamental information for designing targeted therapy and diagnostic biomarkers for TOF.

## Methods

### Study subjects

This was a prospective study performed on fetuses who were identified with TOF by fetal echocardiogram in our hospital between March 2019 and December 2020. The criteria for TOF inclusion were as following: (1) confirmation the diagnosis of TOF, (2) absence of other structural malformations. (3) low risk of cell-free fetal DNA testing and chromosome microarray analysis (CMA) at first trimester. The criteria for controls inclusion were as following: (1) absence of structural malformations, (2) obtainment of myocardial tissues from gestation age-matched fetuses were during the same period were randomly selected as controls [15]. It should be pointed out that the myocardial tissues of TOF and controls were obtained from those that developed spontaneous premature labor or unexplained intrauterine death, and further genetic examination was required by the parents in order to give genentic reference data for their next pregnancy. As VSD and overriding aortic artery were not available for the collection of tissue sample, and the right ventricular hypertrophy was not significant enough in the TOF fetus, so we took stenosis of the pulmonary artery wall and the the corresponding sites were taken in the control group. The protocol was approved by the Ethics Committee of the Affiliated Suzhou Hospital of Nanjing Medical University (KL901184) and all parents signed written informed consents.

#### Fetal ultrasonography

Fetal GA was determined based on the crown-rump length by ultrasound in the first trimester. All fetuses underwent a routine obstetric ultrasound scan to exclude the presence of other intracardiac and extracardiac malformations. Fetal TOF is characterized by the obstruction of right ventricular outflow tract (RVOT), subaortic ventricular septal defect (VSD), and large aorta overriding over both ventricles. Suspected cases with TOF were referred to two highly experienced fetal cardiologists to make the diagnosis in a blind fashion. If a dispute arose, then the final decision was made by a third qualified cardiologist. Ultrasound Equipment.A VolusonTM E8 ultrasound system (GE Healthcare Ultrasound, Milwaukee, WI, USA) coupled with a C1-5-D transducer (frequency 2–5 MHz) was used.

#### Autopsy examination

Unfortunately, spontaneous premature labor or unexplained intrauterine death occurred in a few fetuses with TOF and the controls. With written informed consents from the parents, a detailed autopsy was performed to confirm the ultrasound diagnosis. Myocardial tissue samples were collected and flash frozen in liquid nitrogen for further research.

### Microarray analysis

Microarray analysis for the expression of lncRNAs and mRNAs was performed by Shanghai Gminix Biological Information Company (Shanghai, China), and the accession number for the microarray data reported in this paper is Gene Expression Omnibus database GEO: 184,905. Bioinformatic pipelines for array data analysis were described previously [16].

#### Methods for lncRNA-seq

1. Sample qualification and quantification Total RNA was qualified and quantified as follows: (1) the RNA sample was firstly qualified using 1% agarose gel electrophoresis for possible contamination and degradation; (2) RNA purity and concentration were then examined using NanoPhotometer® spectrophotometer; (3) RNA integrity and quantity were finally measured using RNA Nano 6000 Assay Kit of the Bioanalyzer 2100 system.
2. Library preparation
  RNA library for lncRNA-seq was prepared as rRNA depletion and stranded method.
3. Sequencing
  After library preparation and pooling of different samples, the samples were subjected for Illumina sequencing. Commonly, the lncRNA-seq use PE150 (paired-end 150nt) sequencing for 12G raw data.
4. Quality control for raw data
  Raw data (raw reads) of FASTQ format were firstly processed through in-house perl scripts. In this step, clean data (clean reads) were obtained by removing following reads: (1) reads with 5’ adapter; (2) reads without 3’ adapter or insert sequence; (3) reads with more than 10% N; (4) reads with more than 50% nucleotides with Qphred < = 20; (5) reads with ploy A/T/G/C. Adapter trimming for the removal of adapter sequences from the 3’ ends of reads was also performed. At the same time, Q20, Q30 and GC content of the clean data were calculated. All the downstream analyses were based on the clean data with high quality.
5. Mapping and assembly
  Clean reads for each sample were first mapped to a reference genome with the software HISAT2. Reads alignment results were transferred to the program StringTie for transcript assembly.
6. Identification of lncRNA

All the transcripts were merged using Cuffmerge software. LncRNAs were then identified from the assembled transcripts following four steps: (1) Removal of lowly expressed transcripts with FPKM < 0.5; (2) removal of short transcripts < 200 bp and < 2 exons; (3) removal of the transcripts with protein-coding capability using CNCI, Pfam and CPC2 database; (4) removal of the transcripts mapped within the 1 kb flanking regions of an annotated gene using Cuffcompare. Novel lncRNAs were named following rules of HGNC (The HUGO Gene Nomenclature Committee). The characteristics of novel lncRNA was compared with known lncRNA and mRNA.

#### Methods for mRNA-seq

RNA quantification and qualification ①RNA degradation and contamination was monitored on 1% agarose gels. ② RNA purity was checked using the NanoPhotometer® spectrophotometer (IMPLEN, CA, USA). ③ RNA integrity was assessed using the RNA Nano 6000 Assay Kit of the Bioanalyzer 2100 system (Agilent Technologies, CA, USA). 

Library preparation for Transcriptome sequencing A total amount of 1 µg RNA per sample was used as input material for the RNA sample preparations.

Clustering and sequencing (Genchem Experimental Department) The clustering of the index-coded samples was performed on a cBot Cluster Generation System using TruSeq PE Cluster Kit v3-cBot-HS (Illumia) according to the manufacturer’s instructions. After cluster generation, the library preparations were sequenced on an Illumina Novaseq platform and 150 bp paired-end reads were generated. Data Analysis Quality control Raw data (raw reads) of fastq format were firstly processed through in-house perl scripts.

Reads mapping to the reference genome Reference genome and gene model annotation files were downloaded from genome website directly. Index of the reference genome was built using Hisat2 v2.0.5 and paired-end clean reads were aligned to the reference genome using Hisat2 v2.0.5. We selected Hisat2 as the mapping tool for that Hisat2 can generate a database of splice junctions based on the gene model annotation file and thus a better mapping result than other non-splice mapping tools. Novel transcripts prediction The mapped reads of each sample were assembled by StringTie (v1.3.3b) in a reference-based approach. StringTie uses a novel network flow algorithm as well as an optional de novo assembly step to assemble and quantitate fulllength transcripts representing multiple splice variants for each gene locus.

Quantification of gene expression level feature Counts v1.5.0-p3 was used to count the reads numbers mapped to each gene. And then FPKM of each gene was calculated based on the length of the gene and reads count mapped to this gene. FPKM, expected number of Fragments Per Kilobase of transcript sequence per Millions base pairs sequenced, considers the effect of sequencing depth and gene length for the reads count at the same time, and is currently the most commonly used method for estimating gene expression levels. 

### Statistical analysis

#### Differentially expressed mRNAs and lncRNAs

The abundances of both mRNAs and lncRNAs expression data sets were log-transformed and t-test was then applied to assess the difference in expression between groups. Values were considered statistically significant at *P* < 0.05.

#### Functional annotation of differential mRNAs

For differentially expressed gene abundances, we first generated their corresponding ENSEMBL ids [17]. Next, the ENSEMBL ids of differentially expressed genes were feed into g:Profile [18], a web-based tool to carry out functional pathway enrichment analysis. An association with P < 0.05 was treated as significant.

#### Protein to protein interactions of differential mRNAs

We applied STRING [19] to check if there are potential protein to protein interactions (PPIs) between differential mRNAs. The clustering of PPIs is based on k-means method.

#### Interactions between mRNAs and lncRNAs

The potential interactions between differentially expressed lncRNAs and mRNAs were assessed by using Spearman correlation. An association with P < 0.05 was treated as significant.

#### lncRNAs target mRNA prediction

We applied LncRNA2Target V3 [20] to check if the identified lncRNA-mRNA relationships that show difference between TOF and control have existing experimental confirmations i.e., if certain lncRNA can regulate certain mRNA in experiential sitting.

## Results

### Basic characteristics of participants

There were three fetuses with TOF and three gestation-age matched controls were included in this study. The family histories of all the subjects did not suggest anything out of the ordinary. Cell-free fetal DNA testing of them showed low risks of trisomies 21, 18 and 13. Mean GA at diagnosis was 22.44 week, Mean maternal age was 32.67 years. The results of the genomic microarray analysis suggested normal results and presented in Table 1. There is a clinico-diagnositic flowchart to explain the antenatal management in different cases in supp Fig. 1. Before completion of this manuscript, two of the three families had already welcomed a healthy baby.

**Table 1:** The results of the genomic microarray analysis suggested normal results

Case	Gestation age (weeks)	Cell-free fetal DNA testing (z-score)		
		Trisomy 21	Trisomy 18	Trisomy 13
TOF1	19.28	0.17	-0.27	0.26
TOF2	23.14	0.28	-1.01	-0.71
TOF3	18.84	0.11	0.19	-0.47
Con1	19.42	0.19	0.21	0.27
Con2	23.42	0.31	0.16	0.14
Con3	18.56	0.29	-0.08	-0.28

### Key echocardiographic features of fetal TOF

**Fig. 1:**
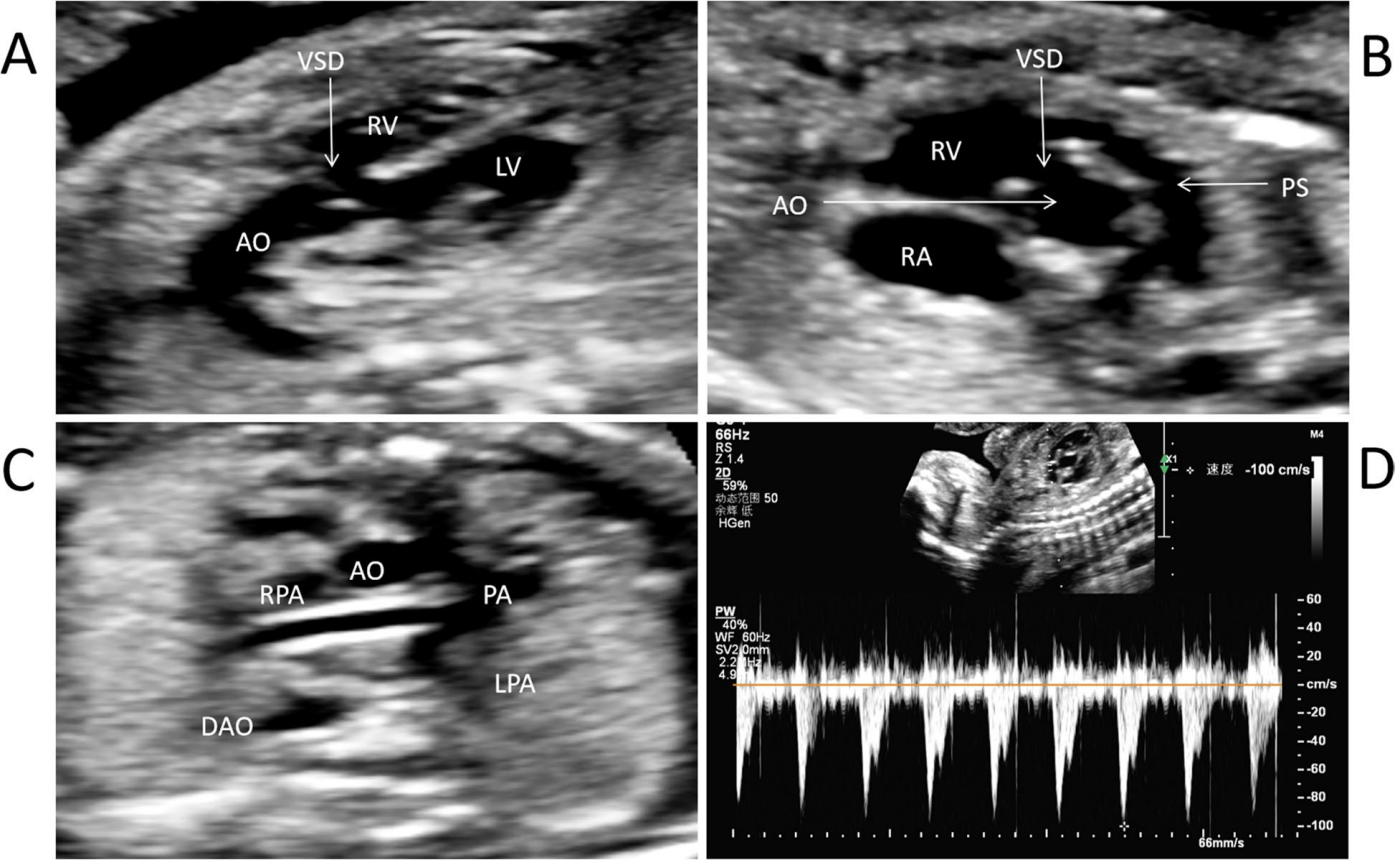
Ultrasonographic observation of fetal TOF. **A** In the left ventricular outflow tract view, a malalignment-type VSD was detected, and the aorta root overrode the ventricular septum. **B** In the short axis view of great arteries, the conal septum moved anteriorly, and stenosis of the RVOT and PA were identified. **C** The diameters of branch pulmonary arteries were slightly small but closed to normal. **D** The typical “double-peak” pattern in systole of spectral Doppler ultrasound in the PA confirmed stenosis of the PA

The key echocardiographic features of fetal TOF was as following: (1)anteriorly deviation of the conal septum, creating the obstruction of RVOT (subpulmonic, pulmonary valve, or supravalvar pulmonary stenosis), (2)large and malalignment subaortic VSD, and (3) large aorta overriding VSD and over both ventricles. To be specific, in the view of the outflow tract of left ventricle, malalignment VSD and overriding aorta was identified, with the overriding rate < 50%. And in the short axis view of great arteries, deviation of the conal septum anteriorly into RVOT and pulmonary stenosis (PS) were confirmed, with the size pulmonary annulus being smaller than the aortic annulus (Fig. 1). In addition, absence of ductus arteriosus, right aortic arch may be associated in some cases.

### Autopsy findings

**Fig. 2:**
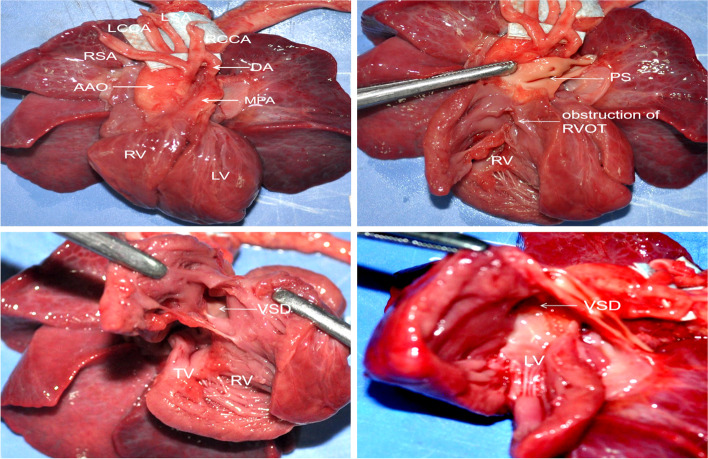
Autopsy findings of fetuses with TOF. **A **The anterior surface view of the fetal heart showed that the PA was significantly smaller than the aorta. Interestingly, the ductus arteriosus connected the PA and the right common carotid artery. **B **Then, the RV and RVOT opened, and a muscular obstruction of the RVOT and a small PA were clearly identified. **C** A perimembranous VSD was observed from the RV, and the root of the aorta overrode the ventricle septum. **D** The LV was opened, and the perimembranous VSD and the overriding of the aorta were confirmed

After careful inspection of the surface of the body and extracardiac organs, the autopsy focused on the heart. To more clearly show the intracardiac structures, both the heart and the lung were removed from the chest. Firstly, from the anterior view of the heart, the PA was significantly smaller than the aorta, which suggested the presence of PS. As we expected, right ventricular hypertrophy was not observed. Secondly, the RVOT was opened from the apex to pulmonary trunk, and the significant small diameter of the PA confirmed the ultrasound diagnosis of PS. Third, the ventricle septum was checked carefully from the right side, and a large perimembranous VSD was found. Through the VSD, the root of the aorta could be easily seen. Lastly, the LV was opened from the left atrium to left ventricle, and the VSD could be seen from the left side. The malalignment of the ventricle septum and the anterior wall of the aorta suggested that the aorta root overrode the ventricular septum. In summary, the autopsy confirmed the prenatal diagnosis of TOF. Interestingly, in one case with TOF, the autopsy demonstrated that the ductus arteriosus connected the PA and the right common carotid artery, which was missed by ultrasound (Fig. 2).

#### Differentially expressed lncRNAs

**Fig. 3:**
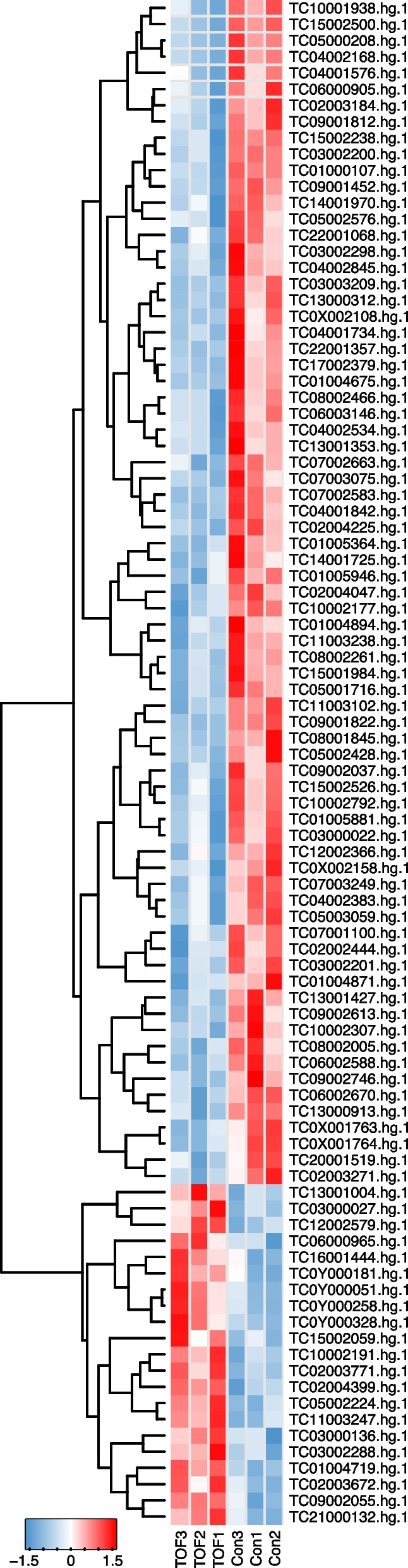
Differentially expressed lncRNAs were shown in the heatmap between the control and fetal TOF group. The darkness of color represents the mean abundance of normalized expression levels across samples between groups

After quality check, we have in total detected 20,473 lncRNAs and 94 of them showed significant differences between the control and fetal TOF group (P < 0.05, Table S1). Upon them, 21 were significantly up-regulated while 73 were significantly down-regulated in fetal TOF group. Standardized expression of differentially expressed lncRNAs were shown in the heatmap (Fig. 3).

#### Differentially expressed mRNAs

**Fig. 4:**
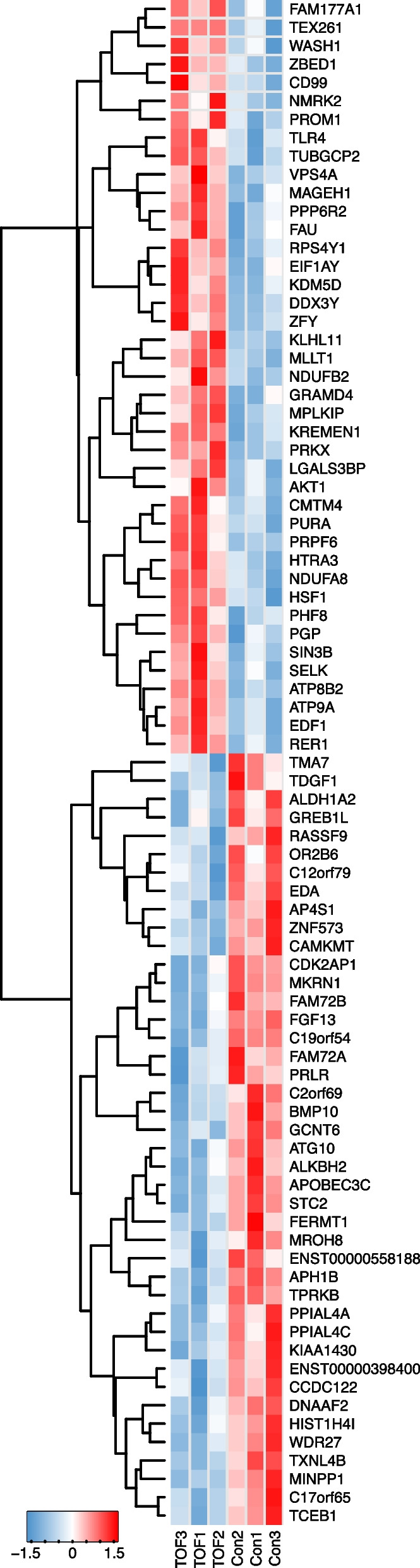
Differentially expressed mRNAs were shown in the heatmap between the control and fetal TOF group. The darkness of color represents the mean abundance of normalized expression levels across samples between groups

In total, 19,371 mRNAs have been detected and compared with the control group, we identified 83 differentially expressed mRNAs, including 41 up-regulated and 42 down-regulated of fetal TOF group (P < 0.05, Fig. 4, Table S2). The most significantly up-regulated and down-regulated mRNA transcripts were MLLT1 and APH1B respectively. We further verified two mRNAs by qPCR and found BMP10 and TDFG1 were down-related in TOF fetus compared with controls, which are consistented with the microarray analysis results (Supp Fig. 2).

#### Functional enrichment of differentially expressed mRNAs

**Fig. 5:**
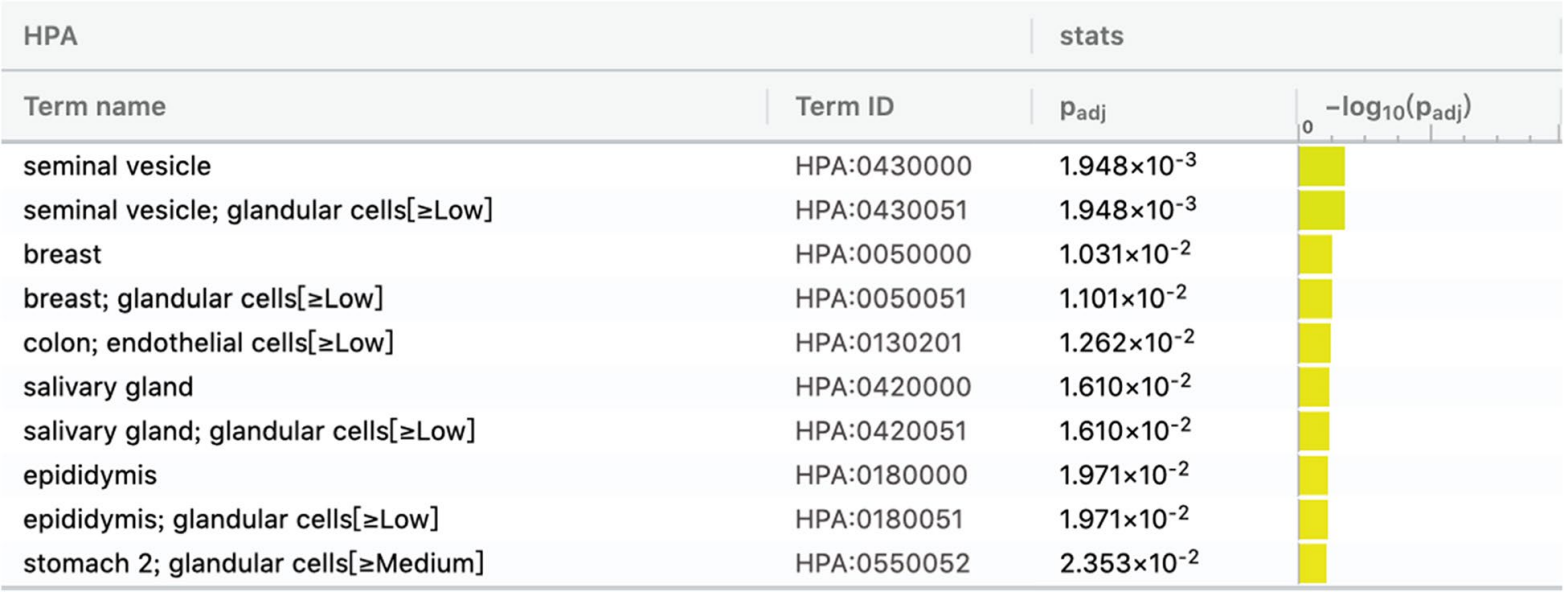
The function of abnormal expression of mRNA showed that some were involved in the pathways of the pathogenesis of many tissues and organs

The function of abnormal expression of mRNA analyzed by software showed that some involved in the pathways of the pathogenesis of many tissues and organs, such as seminal vesicle, glandular cells, breast, colon, endothelial cells, salivary gland, glandular cells, epidermis, stomach. The mRNAs included MLLT1、ATP8B2、APOBEC3C、PRKX、DDX3Y、TUBGCP2、NDUFA8、HTRA3、EDF1 and et al. (Fig. 5).

#### Protein to protein interactions between differentially expressed mRNAs

**Fig. 6:**
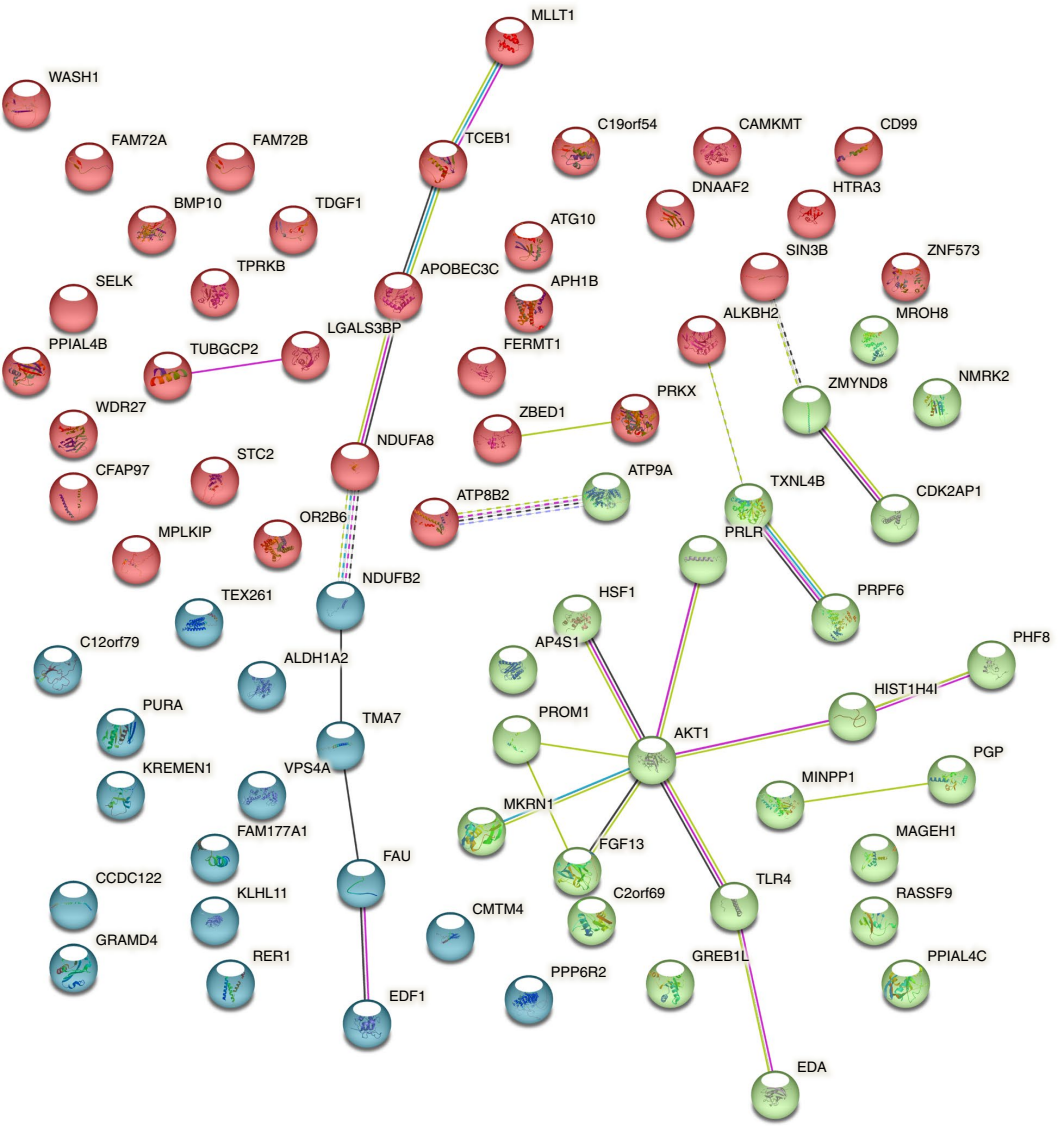
Protein to protein interactions between differential expressed mRNAs. Different colors represent protein to protein interaction clusters by using k-means method

By using STRING to check potential protein to protein interactions (PPI) between differential mRNAs, we showed that there were three PPI clusters (Fig. 6). Suggesting some proteins that may jointly contribute toward a specific cellular process that may important for TOF.

#### Potential interactions between lncRNAs and mRNAs

Correlation analysis of lncRNAs and mRNAs showed that differentially expressed lncRNAs were closely related to mRNAs (Table S3). We found positive correlations of lncRNAs and mRNAs, including TCONS_00005490-XLOC_002569 and ATP8B2, n324056 and PPIAL4A, ENST00000415883 and NDUFB2, ENST00000445967 and TLR4, TCONS_00021936-XLOC_010529 and MAGEH1, n339687 and EDA, NR_038883 and ZFY, NR_001545 and DDX3Y, NR_001545、NR_038883 and KDM5D, n338336 and C17orf65. More interestingly, we found negative correlations of lncRNAs and mRNAs, including TCONS_00003990-XLOC_001813 and PPIAL4A, TCONS_00014231-XLOC_006288 and PRLR, n338858 and WDR27, n326323 and EDF1, ENST00000412266 and LGALS3BP.

We further checked if there were existing experimental evidences to confirm the reported lncRNA-mRNA relationships with LncRNA2Target V3. However, we didn’t find any existing evidences, suggesting that our results might be novel, but further experimental validations are essential to confirm those results.

## Discussions

Fetal TOF is a conotruncal malformation and is commonly seen in the clinic. It is not difficult to detect and identify fetal TOF via fetal echocardiogram, especially in the second and third trimesters. In fact, an increasing number of cases are identified during the first trimester. The degree of PS is the determining factor for predicting the prognosis of fetal TOF in vivo. If possible, autopsy allowed for a better understanding of all the anatomic abnormalities in fetal TOF, facilitating an improvement of the prenatal diagnostic rate and furthering basic research.

Some researchers have found that differences of lncRNAs could be detected in the body fluids of patients with TOF, which is effective evidence for the further study of lncRNA as a biomarker of TOF [21]. Zhang X et al. found that four hub lncRNAs that regulate mRNA expression through miRNAs in heart tissues of 22 children with TOF [22]. Jing Ma et al. found lncRNA TBX5-AS1:2 was involved in TOF and Quan Wang et al. reported that lncRNA HA117 was expressed increasingly and caused to adverse short-term outcomes from TOF patients [11, 12].

More deeply, we detected lncRNAs and mRNAs in myocardial tissues of fetal TOF and control groups and found the 94 differentially expressed lncRNAs, which could be very important for the development of fetal TOF and be candidate for early prevention and intervention. Currently, the roles of theses abnormally expressed lncRNAs in TOF are not reported. We found 83 abnormally expressed mRNAs in fetal TOF compared with normal controls, which could be target gene to participate in the pathological process of fetal TOF. At present, there are only two abnormally expressed mRNAs in fetal TOF detected by us are reported in heart diease, which are PURA and ALDH1A2. Marilene Pavan et al. reported that the genetic variation of ALDH1A2 was present in TOF, but not a significant risk of congenital heart disease [23]. Miriam S. Reuter reported that recurrent variants p.(Phe271del) in PURA leaded to structural heart defects [24].

More importantly, we constructed the correlation analysis of lncRNAs and mRNAs from the both abnormally expressed lncRNAs and mRNAs. It is more meaningful for negative correlation of lncRNAs and mRNAs, and n326323 and endothelial differentiation-related factor 1 (EDF1) are important one couple. The function of lncRNA n326323 is not reported at present, and it might be important in heart development and lead to fetal TOF. For the target gene of lncRNA n326323, EDF1 was reported as a stem-cell-like gene in early development of endothelial cells, highly expressed in MSC [25]. EDF1 was also a protein that could regulate an immediate-early transcriptional response, and was a ZNF598-independent sensor, stabilizing GIGYF2 to inhibit translation initiation [26, 27]. Furthermore, EDF1 was a transcriptional coactivator of PPARγ, and was required for VEGF-induced transcriptional activity of PPARγ in human endothelial cells [28]. EDF1 initiated the activation of lipogenic gene program cooperated with lncRNA Blnc1, which suggested lncRNA could interacte with EDF1 [29]. These indicated that lncRNA n326323 might negatively regulate EDF1 gene, which played an important role in the development of heart.

Prolactin receptor (PRLR) is another candidate gene that could be negatively regulated by lncRNA TCONS_00014231-XLOC_006288. PRLR gene was important for the reproduction and growth [30]. The silencing of PRLR gene leaded to the inhibition of hippocampal neuron apoptosis, which indicated the role of PRLR in cell apoptosis [31]. And PRLR protein expression was increased in hypertrophy hearts, and involved in the development of cardiac hypertrophy [32]. PRLR was also a gene (PRLRa) which played an important role for the regulation of osmotic and related to the abnormal renal development [33]. LGALS3BP is another candidate gene that could be negatively regulated by lncRNA ENST00000412266, which plays a role in lipid metabolism and other different pathways [34]. The protein expression of LGALS3BP was upregulated in leukocyte migration and invasion, and involved in the progression of inflammation [35]. Recently study reported that LGALS3BP protein could interact with SARS-CoV-2 spike glycoprotein, which is an important clue for its function in the treatment of SARS-CoV-2 [36].

WDR27 is another candidate gene that could be negatively regulated by lncRNA n338858. WDR27 was reported to be cilia/basal body localization and participated in the evolution of ciliary processes [37]. WDR27 was loci associated with insomnia symptoms (in males) [38], included in the third most significantly associated SNP of type 1 diabetes [39], and was a genome-wide significant loci associated with eye shape [40]. PPIAL4A is a very interesting gene that could not only be negatively regulated by TCONS_00003990-XLOC_001813, but also positively regulated by lncRNA n324056, and its function is unknown, which is worth furthermore research. At present, the roles of these couples of mRNAs and lncRNAs are not reported in fetal TOF, which are needed to explore.

In summary, fetal TOF was prenatally diagnosed by fetal echocardiogram and confirmed by pathological observations. LncRNAs and mRNAs expression profiles were used in this study to screen out the lncRNAs related to fetal TOF and the related function involved, but whether they are specific lncRNAs for TOF needs to be verified by more cases and further confirmed by in vitro and in vivo experiments. These abnormal lncRNAs screened in fetal TOF heart tissues are promising as molecular markers or gene therapy targets for early diagnosis and treatment of fetal TOF. We also acknowledge that due to the limited samples size, the reported differential lncRNAs and mRNAs in TOF still need confirmation either by further studies.

### Conclusion

- No significant changes were found in DNA level of TOF fetus, but the characterization of TOF was found by ultrasound and autopsy. Therefore, we further conducted microarray sequencing to find the pathogenic cause of TOF from mRNA and lncRNA levels.
- LncRNAs and mRNAs expression profiles were used in this study to screen out the lncRNAs/mRNAs related to fetal TOF and the related function involved.
- These abnormal lncRNAs and mRNAs screened in fetal TOF heart tissues are promising as molecular markers or gene therapy targets for early diagnosis and treatment of fetal TOF.

## Supplementary Information

**Additional file 1: Supp Fig. 1.** A clinico-diagnositicflowchart of the antenatal management in different cases. **SuppFig. 2.** The differential  expression mRNAswere verified by RT-PCR in TOF patients and Controls.

**Additional file 2:** **Table S1. **Differential lncRNAs between TOF and control groupincluding 21 up- and 73 down-regulated lncRNAs. **Table S2. **Differential mRNAs between TOF and control groupincluding 41 up- and 42 down-regulated genes. **Table S3. **lncRNA correlations to mRNA showed difference betweenTOF and control group.

## Data Availability

The accession number for the microarray data reported in this paper is Gene Expression Omnibus database GEO: 184,905. The web links are https://www.ncbi.nlm.nih.gov/geo/query/acc.cgi?acc=GSE184905. Enter token efwbsyaextyjbqh into the box.
