# Supplementary material for: Prenatal diagnosis and mRNA profiles of fetal tetralogy of Fallot
Source: BMC Pregnancy Childbirth. 2022 Nov 19;22:853. doi: 10.1186/s12884-022-05190-0 (PMC9675103; doi:10.1186/s12884-022-05190-0)
Supplement: Supplementary file 1 — Additional file 1: Supp Fig. 1. A clinico-diagnositicflowchart of the antenatal management in different cases. SuppFig. 2. The differential expression mRNAswere verified by RT-PCR in TOF patients and Controls. [file 12884_2022_5190_MOESM1_ESM.pptx]

## Slide 1
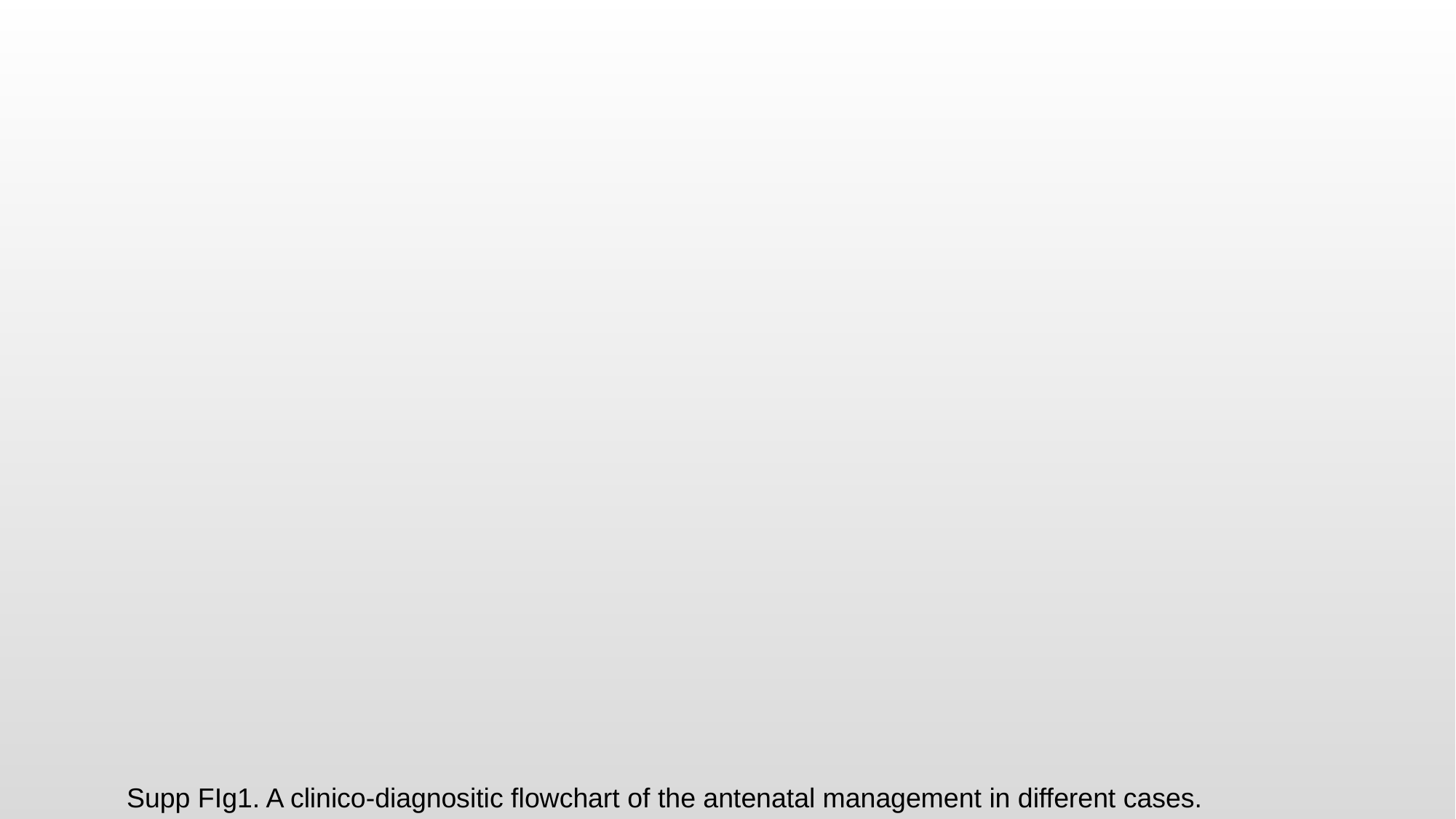

Supp FIg1. A clinico-diagnositic flowchart of the antenatal management in different cases.

## Slide 2
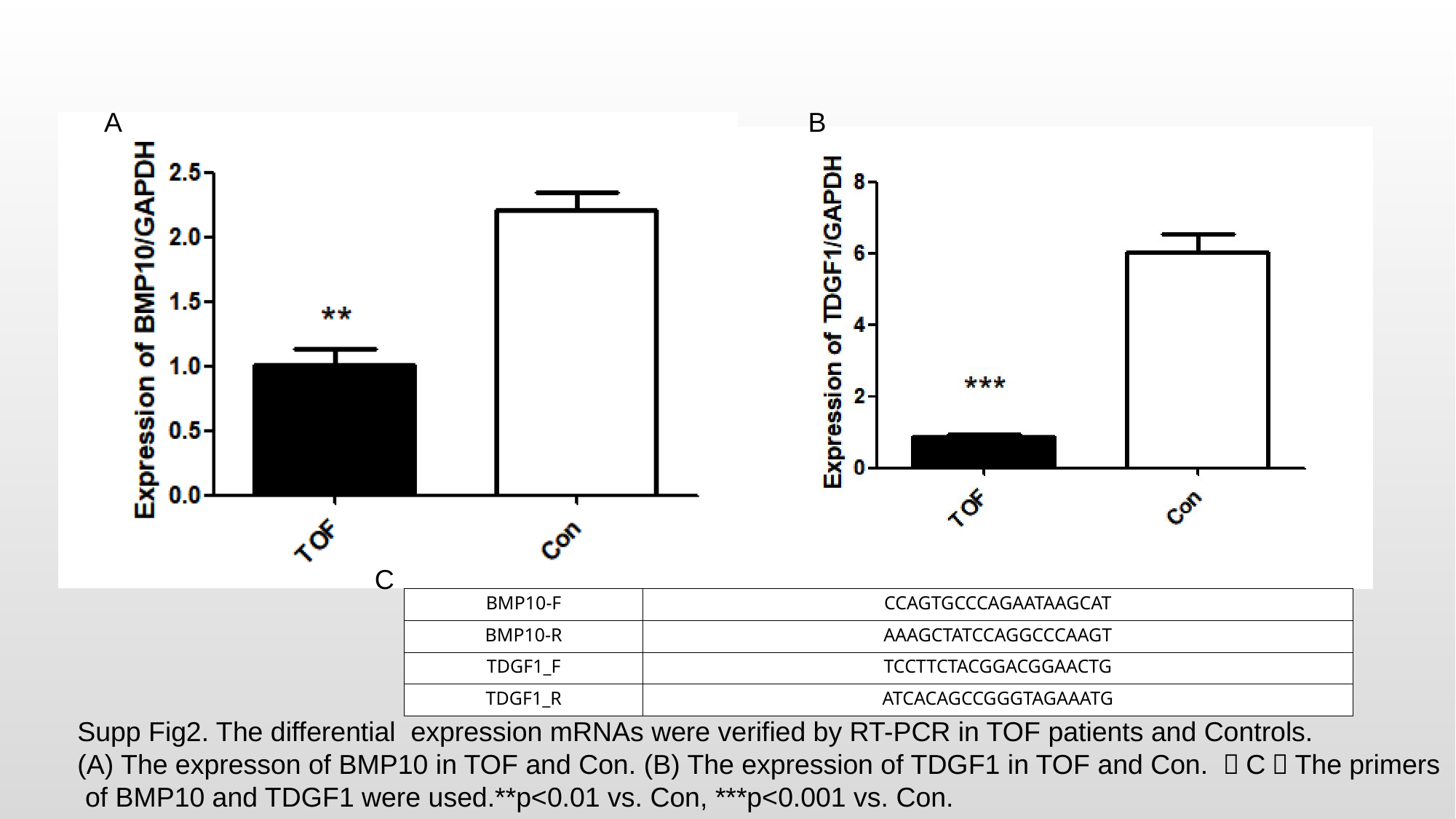

A
B
C
| BMP10-F | CCAGTGCCCAGAATAAGCAT |
| --- | --- |
| BMP10-R | AAAGCTATCCAGGCCCAAGT |
| TDGF1\_F | TCCTTCTACGGACGGAACTG |
| TDGF1\_R | ATCACAGCCGGGTAGAAATG |
Supp Fig2. The differential expression mRNAs were verified by RT-PCR in TOF patients and Controls.
(A) The expresson of BMP10 in TOF and Con. (B) The expression of TDGF1 in TOF and Con. （C）The primers
 of BMP10 and TDGF1 were used.**p<0.01 vs. Con, ***p<0.001 vs. Con.
